# Exploring the Effects of Yoga on the Resting Metabolic Rate and Fat-to-Muscle Ratio of Medical Students: A Six-Week Interventional Study

**DOI:** 10.7759/cureus.85314

**Published:** 2025-06-04

**Authors:** Pooja Prajapati, Preeti Kaliramana, Fariza Jamil, Radhika Agarwal, Prerna Agarwal, Bharti Bhandari

**Affiliations:** 1 Physiology, Government Institute of Medical Sciences, Greater Noida, IND; 2 Physiology, Noida International Institute of Medical Sciences, Greater Noida, IND; 3 Physiology, Saraswati Medical College, Unnao, IND

**Keywords:** body mass index, fat-to-muscle ratio, medical students, resting metabolic rate, yoga

## Abstract

Introduction

There is a growing body of research examining the effects of yoga on health outcomes; however, limited research has focused specifically on its impact on resting metabolic rate (RMR) and body composition. Understanding how yoga training influences these parameters in healthy young adults could provide valuable insights into the potential mechanisms underlying the health benefits of yoga. This study was planned to investigate the effect of short-term (six weeks) yoga training on RMR and the fat-to-muscle ratio in healthy young adults.

Methods

This randomized controlled trial was initiated after registration with the Clinical Trial Registry of India (CTRI) and obtaining approval from the Institutional Ethics Committee. Written informed consent was obtained from all participants. Fifty apparently healthy medical students, between 20 and 25 years of age and fulfilling the inclusion criteria, were randomly assigned to the “Yoga Group” or the “Control Group.” The Yoga Group received yoga training according to a standard protocol for six weeks. Weight, body mass index (BMI), RMR, and fat-to-muscle ratio were measured at baseline and after six weeks of yoga training using a body composition analyzer.

Results

No significant difference was observed in RMR or any of the anthropometric measures between the Control Group (without yoga) and the Yoga Group after six weeks of yoga training. However, the mean change (baseline to six-week value) was significantly greater in the Yoga Group compared to the Control Group for weight (0.20 ± 2.25 vs. -0.68 ± 0.70) and BMI (0.17 ± 2.26 vs. -0.82 ± 1.11).

Conclusion

No significant improvements were observed in RMR or fat-to-muscle ratio following six weeks of yoga; however, minor changes in weight and BMI suggest possible longer-term effects. To achieve a change in fat-to-muscle ratio and to induce alterations in resting and basal metabolic rate, a more rigorous (e.g., power yoga) and prolonged, regular regimen of yoga training may be required.

## Introduction

Resting metabolic rate (RMR), also called resting energy expenditure, is defined as the energy required by the body in a resting condition, when the individual is awake, in a postabsorptive, thermoneutral state, and has not exercised for typically 12 hours [1]. It plays a significant role in energy balance and weight management. Various factors influence RMR, including body composition, age, gender, and physical activity level [1,2]. Yoga, an ancient practice originating in India, has gained popularity worldwide due to its numerous health benefits.

Different procedures practiced in yoga have stimulatory or inhibitory effects on the body’s energy expenditure. Studies have shown that controlling the breathing pattern during yoga can alter the oxygen consumption by the body, which will further help in maintaining the fat-to-muscle ratio [3-5]. Fat-to-muscle ratio has been proposed as an alternative approach for assessing body fat, and is a good predictive indicator of metabolic syndrome [6]. Maintaining a healthy fat-to-muscle ratio involves a balanced diet, regular physical activity, and strength training exercises. The body fat percentage has been shown to reduce significantly by practicing different yogic exercises, thus maintaining an appropriate fat-to-muscle ratio and weight [7,8].

While there is a growing body of research examining the effects of yoga on health outcomes, limited research has focused specifically on its impact on RMR and body composition. Understanding how yoga training influences these parameters in healthy young adults could provide valuable insights into the potential mechanisms underlying the health benefits of yoga. Several studies have demonstrated that long-term yoga practice can help regulate risk factors associated with cardiovascular disease, obesity, and overall body composition, including muscle and fat content [7,8]. Yogic postures and breathing exercises have the potential to influence RMR and improve the fat-to-muscle ratio, thereby contributing to the management of these conditions.

Due to their busy schedule and academic pressure, most medical students are not very actively engaged in physical activities, which may predispose them to weight gain and other health issues [9]. Yoga is a form of physical activity that has proven to be beneficial in improving metabolism and reducing cardiovascular complications. There is a paucity of studies examining the effect of yoga on RMR and fat-to-muscle ratio among young students. Hence, the present study is planned to assess the effect of short-term yoga (asanas, pranayama, and meditation) on RMR and fat-to-muscle ratio in apparently healthy young medical students.

## Materials and methods

The study commenced after obtaining approval from the Government Institute of Medical Sciences-Institutional Ethics Committee, Greater Noida, India (approval no. GIMS/IEC/HR/2023/31). The trial was registered under the Clinical Trial Registry of India (CTRI/2024/01/061004). Written informed consent was obtained from all the participants.

Study design, population sample, and randomization

It was a randomized, controlled, open-label trial involving young medical students of both genders, between 20 and 25 years of age. 

The sample size was calculated using the given values of body fat mass in the two groups from the study by Na Nongkhai et al. [8]. With a significance level of 0.05, power (1-β) of 80%, ratio of sample size (treatment/control) as 1, allowable difference of 2.65, standard deviation of 3.47, and dropout rate of 10%, the total sample size was 26 (13 in each group). More students were interested in participating; hence, 60 students fulfilling the inclusion criteria were enrolled in the study (30 in each group). Finally, 25 students in the Yoga Group who completed at least 80% of the yoga sessions, and 25 in the Control Group, were included.

Young, apparently healthy students from a medical college located in Western Uttar Pradesh (age group 20 to 25 years), with a normal body mass index (BMI between 18 kg/m² and 25 kg/m²), and who consented to participate, were included in the study.

Individuals involved in any regular exercise, sports, yoga, or meditation; those with any physical disability that prevents them from performing breathing exercises or yoga postures; those with any major physical, systemic, or psychiatric illness; and those on any regular medication were excluded. Chronic smokers or alcoholics (as per WHO guidelines), and individuals who use any stimulants or recreational drugs were also excluded.

The included participants were randomized into two groups of 25 each using a computer-generated random number sequence, as follows: (1) Yoga Group - subjects received supervised yoga training for 60 minutes per day, conducted by a trained yoga expert in the evening at 6 PM, every day of the week. The yoga sessions were conducted in the presence of residents from the department to ensure compliance and attendance. (2) Control Group - subjects were not given any yoga training.

Recorded parameters in each group

Physiological Parameters

The physiological parameters included height, weight, and BMI. BMI was calculated by dividing weight (kg) by the square of height (m) (Quetelet’s Index).

RMR and Muscle-to-Fat Ratio Measurement

The parameters were measured at baseline and after six weeks of yoga training. Body composition measurement was done using a body composition analyzer (InBody 570; InBody USA, Cerritos, CA, USA), based on bioelectrical impedance analysis (BIA). The participants were instructed not to have a meal three to four hours prior to the test; to avoid beverages containing caffeine or other beverages at least four hours before measurement; and not to engage in exercise or activities that cause heavy sweating. For the measurement, the subjects were asked to sit quietly for 15 minutes. This time is important for the subjects to relax and allow their metabolism to decrease toward a resting state. Before recording, the participants were asked to take off their socks or stockings and place their bare feet firmly on the plate electrodes. The participants were instructed to grip the handle electrodes with their fingers and palms, stretch both arms, and spread them 30° from the body. The participants were instructed not to speak or move their bodies until the measurement was completed. Upon completion, the result was displayed on the screen and printed out on a pre-printed result sheet.

Yoga protocol

The protocol given by Chaya et al. was followed in the study as given below [3]. The yoga training was delivered by a trained yoga practitioner in the presence of residents from the department. A mixed set of yoga techniques, in the form of asana (postures), deep relaxation technique, pranayama (breathing techniques), and meditation, were practiced daily for an hour, for a period of six weeks. The asana postures were started with stretching techniques, followed by standing, supine, prone, and sitting postures. The standing postures included side bending triangle posture (trikonasana), forward bending (padahastasana), backward bending (ardha chakrasana), and side lateral bending (ardhakati chakrasana) techniques. The supine postures practiced were straight leg raising and shoulder stand posture (sarvangasana); the prone postures were locust (shalabhasana), serpent (bhujangasana), and bow (dhanurasana) postures. The sitting postures included the moon (shasankasana), hardy (vajrasana), and the half matsyendra (ardha matsyendra) postures. The asanas were followed by a deep relaxation technique, which was performed for six minutes with closed eyes, with specific instructions relating to awareness and relaxation of different parts of the body. The pranayama included kapalabhathi, shithali, shithkari, vibhagiya, and nadishuddhi pranayama. This session ended with meditation and shavasana. 

Statistical analysis

Data was analyzed using GraphPad Prism version 8.0.0 for Windows (GraphPad Software, San Diego, CA, USA). Data was tested for normality and expressed as mean ± standard deviation. For comparison between pre- and post-test values, a t-test for dependent samples was used. Mean difference (%) between pre- and post-test values in the two groups was calculated and analysed using a t-test for independent samples. A significance level of p < 0.05 was used in the study.

## Results

The mean age (in years) in the Control Group and Yoga Group was 21.72 ± 1.59 and 20.24 ± 1.79, respectively. In the Control Group, out of 25 participants, 72% (18) were males, whereas in the Yoga Group (n = 25), 60% (15) were males.

The anthropometric parameters recorded in the two groups are given in Table 1. The table also depicts the percent change in all the parameters in the two groups. No significant difference was found between the baseline and post-six-week values in the Control Group as well as the Yoga Group; however, the mean change between the Control Group and Yoga Group was found to be significant for weight and BMI.

**Table 1:** Anthropometric parameters and RMR in the Control Group and Yoga Group *p < 0.05 was considered statistically significant. Data is presented as mean ± SD. The mean change (%) was calculated using the formula: (Final Value - Initial Value) ÷ (Initial Value) × 100. Intergroup comparison was done using a t-test for dependent samples. The mean difference between pre- and post-values in the two groups was calculated and analysed using a t-test for independent samples. BMI: body mass index; SMM: skeletal muscle mass; RMR: resting metabolic rate; WHR: waist-hip ratio

Parameters	Control Group (n = 25)		p-value	Mean Change (%)	Yoga Group (n = 25)		p-value	Mean Change (%)	p-value (Mean Change)
	Baseline	At 6 weeks			Baseline	At 6 weeks			
Height (cm)	165.7 ± 8.8	165.7 ± 8.8	-	-	156.8 ± 6.1	156.8 ± 6.1	-	-	-
Weight (kg)	60.8 ± 11.7	61.2 ± 11.8	0.68	-0.68 ± 0.7	57.14 ± 9.6	56.9 ± 8.8	0.61	0.20 ± 2.25	0.03*
BMI (kg/m^2^)	22.1 ± 3.3	22.4 ± 3.3	0.65	-0.82 ± 1.1	23.13 ± 2.8	23.1 ± 2.7	0.78	0.17 ± 2.26	0.02*
SMM (kg)	24 ± 5.8	24.1 ± 5.8	0.81	-0.29 ± 1.0	19.01 ± 2.6	19 ± 2.6	0.91	0.31 ± 2.14	0.06
Body fat mass (kg)	17.1 ± 7.9	17.1 ± 7.9	0.70	-0.58 ± 2.0	21.4 ± 5.8	21.4 ± 5.3	0.87	-0.64 ± 7.07	0.48
Fat-muscle ratio	0.8 ± 0.40	0.8 ± 0.4	0.78	-0.30 ± 2.3	1.1 ± 0.2	1.1 ± 0.2	0.9	-1.04 ± 8.02	0.33
RMR (Kcal)	1312 ± 204.5	1313 ± 205	0.83	-0.11 ± 0.9	1141 ± 97.3	1138 ± 96	0.69	0.25 ± 1.77	0.18
WHR	0.9 ± 0.1	0.9 ± 0.1	0.34	-1.48 ± 3.8	0.9 ± 0.1	0.9 ± 0.1	0.91	-2.44 ± 3.9	1.15
Visceral fat	7.6 ± 4.5	7.8 ± 4.5	0.79	-7.15 ± 16.2	10.52 ± 3.6	10.32 ± 3.2	0.27	-0.19 ± 14.3	0.06

## Discussion

The study aimed to assess the changes in RMR and fat-to-muscle ratio in healthy young adults after six weeks of yoga training. In addition, BMI, waist-hip ratio, and visceral fat were also measured. The study did not reveal any significant outcome; however, the percent change in weight and BMI (baseline and after six weeks) in the Yoga Group was significantly more than the percent change observed in the Control Group. No significant change was observed in terms of skeletal muscle mass, body fat mass, and RMR. 

Extensive work has been done on the effect of long-term and short-term yoga on weight and other anthropometric parameters. Varied, contradictory conclusions have been drawn. As per a systematic review on the effect of yoga on anthropometry, due to poor designs and evidence in the published literature, it is inconclusive to say that yoga can significantly improve anthropometric parameters [10]. Our study did not show any change in muscle mass or body fat percentage. Na Nongkhai et al. postulated that continuous yoga practice is associated with a significant decrease in BMI and body fat mass only at the 12th week, not at the 8th week, whereas a significant increase in muscle mass was observed even at the 8th week, showing the importance of continuous yoga practice [8]. The possible reason for not observing any change in these parameters in our study was the short duration (six weeks) of yoga training for the students. The concept of fat-to-muscle ratio has emerged as a new method for evaluating body fat levels. It serves as a reliable predictor of metabolic syndrome. Achieving and sustaining a favorable fat-to-muscle ratio necessitates adopting a well-rounded lifestyle, which includes maintaining a balanced diet, engaging in consistent physical activity, and incorporating strength training routines. Through conscientious monitoring and fine-tuning of body composition, people can bolster their overall well-being, elevate athletic prowess, and mitigate the likelihood of chronic ailments [6]. A literature search could not retrieve any articles on the effect of yoga on the fat-to-muscle ratio. In this study, we did not find any significant change in this parameter.

The study did not reveal any significant change in weight or BMI in the groups; however, when the percent fall in weight and BMI in the Yoga and Control Groups was compared, it was found to be significantly more in the Yoga Group. As per a systematic review and meta-analysis published in 2016, most of the yoga trials in healthy adults showed no effects on weight, BMI, body fat percentage, or waist circumference. However, in studies including overweight or obese participants, a significant reduction in weight and BMI was observed in the Yoga Group when compared to the Control Group [11]. Similarly, a 12-week yoga combined with aerobic training program caused a significant fall in BMI in overweight and obese college students, but no change was observed in normal-weight students [12]. Contrary to this, Pandit et al. concluded that even a one-week-long yoga camp, if associated with a lacto-vegetarian diet and restriction on sugar, salt, and fat intake, can significantly reduce weight, body fat mass, body fat percentage, and BMI [7]. Yoga seems to be a fitting and potentially effective approach for maintaining weight, preventing obesity, and reducing the risk of diseases where obesity is a significant factor in causation.

We did not observe any change in RMR after six weeks of yoga training. The research conducted by Chaya et al. demonstrated a notable decrease in basal metabolic rate (BMR) associated with prolonged engagement in yoga, likely connected to diminished arousal. This effect was observed through the utilization of a blend of both stimulating and inhibitory yogic techniques [3]. Other studies have demonstrated an increase in metabolic rate and energy expenditure in certain types of yoga [3,5,13]. The observed increase in RMR in these studies may be attributed to various factors, including enhanced muscle mass, improved insulin sensitivity, and reduced stress levels associated with yoga practice [5,13]. Another systematic review concluded that only scant evidence supports the enhancement of energy intake and energy expenditure in adults with overweight or obesity through yoga; the results from these studies are also contradictory [14]. Hagins et al. examined and compared the metabolic costs of yoga across sessions to the metabolic costs of aerobic forms of physical activity. They concluded that only certain high-intensity forms of yoga practice are capable of altering energy expenditure during the session; others do not meet recommendations for levels of physical activity for improving or maintaining health or cardiovascular fitness [15].

Abdominal obesity is a major risk factor for morbidity and mortality. Six weeks of yoga training could not reduce the waist-hip ratio and visceral fat among yoga-practicing healthy students [16]. However, a prior published study postulated that in women with abdominal obesity (waist circumference ≥ 88 cm; BMI ≥ 25), supervised yoga sessions with a mean duration of 30.2 ± 9.2 hours (maximum, 42) significantly reduced abdominal circumference [17]. The efficacy of yoga in facilitating weight loss correlates with several crucial factors, including heightened practice frequency, extended intervention duration, and the inclusion of a yogic dietary component. Furthermore, researchers have proven that the benefits of regular yoga practice are much more evident in individuals who are overweight or obese.

Limitations such as small sample size, limited study duration, and certain methodological constraints must be acknowledged. Future studies should consider a larger sample, extended duration, and a more intensive yoga intervention for more robust outcomes. Key strengths of the study include its design, clearly defined population, and comprehensive data collection.

## Conclusions

The findings of this study suggest that six weeks of yoga training is not adequate to bring about beneficial changes in RMR and body composition in healthy young adults. No significant difference in the baseline and six-week values of any of the parameters was observed in the Yoga Group. However, a significant mean difference (percent change) between baseline and after six weeks in weight and BMI in the Yoga Group, when compared to the Control Group, was recorded. This may be suggestive of the potential impact of consistent yoga practice on weight reduction. Six weeks of yoga did not significantly alter RMR or body composition among healthy medical students. Future studies with longer durations and larger sample sizes are needed to explore the potential metabolic effects of yoga. 
